# Maximizing the Quality and Reporting Standards of Autism Intervention Science

**DOI:** 10.1002/aur.70126

**Published:** 2025-10-10

**Authors:** Shannon LaPoint, Claire Brito Klein, Micheal Sandbank, Kristen Bottema‐Beutel, Sue Fletcher‐Watson, Gauri Divan, Dagmara Dimitriou, Evdokia Anagnostou, Mette Elmose Andersen, Amanda Binns, Tony Charman, Jasper A. Estabillo, Stephanie M. Fecteau, Anna Ferrari, Marie‐Maude Geoffray, Lauren H. Hampton, Sabri Hergüner, Emily S. Kuschner, Jia Ying Sarah Lee, Julie Segers, Deanna Swain, Sarah Vejnoska, Giacomo Vivanti, Chongying Wang, Jonathan Green

**Affiliations:** ^1^ School of Teacher Education Florida State University Tallahassee Florida USA; ^2^ TEACCH Autism Program University of North Carolina at Chapel Hill Chapel Hill North Carolina USA; ^3^ School of Medicine University of North Carolina at Chapel Hill Chapel Hill North Carolina USA; ^4^ Lynch School of Education and Human Development Boston College Chestnut Hill Massachusetts USA; ^5^ Salvesen Mindroom Research Centre University of Edinburgh Edinburgh UK; ^6^ Sangath Badez Goa India; ^7^ Psychology and Human Development University College London London UK; ^8^ Holland Bloorview Kids Rehabilitation Hospital University of Toronto Toronto Ontario Canada; ^9^ Department of Psychology University of Southern Denmark Odense Denmark; ^10^ Department of Speech‐Language Pathology University of Toronto Toronto Ontario Canada; ^11^ School of Communication Sciences & Disorders Western University London Ontario Canada; ^12^ Institute of Psychiatry, Psychology & Neuroscience King's College London London UK; ^13^ Semel Institute for Neuroscience & Human Behavior University of California Los Angeles California USA; ^14^ Department of Psychoeducation and Psychology Université du Québec en Outaouais Saint‐Jerome Quebec Canada; ^15^ Department of Psychology University of Milano Bicocca Milan Italy; ^16^ RESHAPE, INSERM U1290 Lyon France; ^17^ Université Claude‐Bernard Lyon 1 Lyon France; ^18^ CH Le Vinatier Bron France; ^19^ The University of Texas at Austin Austin Texas USA; ^20^ Ankara Autism Center Ankara Türkiye; ^21^ Children's Hospital of Philadelphia Philadelphia Pennsylvania USA; ^22^ Department of Psychiatry, Perelman School of Medicine University of Pennsylvania Philadelphia Pennsylvania USA; ^23^ Queensland Cerebral Palsy and Rehabilitation Research Centre, UQ Child Health Research Centre The University of Queensland Brisbane Queensland Australia; ^24^ Faculty of Psychology and Educational Sciences KU Leuven Leuven Belgium; ^25^ Department of Pediatrics University of Colorado School of Medicine Aurora Colorado USA; ^26^ Department of Psychiatry and Behavioral Sciences University of California, Davis MIND Institute Sacramento California USA; ^27^ AJ Drexel Autism Institute, Drexel University Philadelphia Pennsylvania USA; ^28^ Autism Research Center, School of Sociology Nankai University Tianjin China; ^29^ Division of Psychology and Mental Health University of Manchester Manchester UK; ^30^ Royal Manchester Children's Hospital Manchester UK

**Keywords:** autism, INSAR, intervention, reporting standards, special interest group, trial

## Abstract

Although there are clear international standards for intervention science and reporting in healthcare, implementation and uptake have been limited within autism intervention research. To address this concern, a Special Interest Group (SIG) was convened at the International Society for Autism Research (INSAR) Annual Meetings in May 2023 and May 2024. This SIG comprised members of the autistic community, senior clinical scientists, clinicians, advanced researchers, and early career researchers, who discussed and debated quality standards for autism intervention trials. This commentary summarizes relevant literature highlighted by SIG panelists and recommendations generated from small breakout groups and larger group discussions with SIG attendees. We recommend that all journals publishing autism intervention findings, especially autism‐focused journals, institute mandatory reporting practices (e.g., trial registration, protocol, analysis plan) to facilitate transparency and rigorous autism intervention science, as well as related education initiatives in support of this goal. Findings from the SIG offer practical, actionable recommendations that we advocate be systematically adopted across autism‐focused journals.

**Summary**

- In recent years, there have been critiques about the quality of autism intervention research.
- Autistic people and their families deserve high‐quality, evidence‐based information about the services available to them, and this information must be reported clearly.
- This is not always the case. Therefore, various community members with lived experience and/or expertise in carrying out research and writing reports (e.g., clinicians and researchers) met at an international conference to discuss these issues.
- People who attended the group suggested recommendations for journals that publish autism studies and related organizations that can offer training and insist on or encourage best practices for studies to improve autism intervention research.

## 1 Introduction

In recent years, researchers have expressed concerns about the quality of the autism intervention science literature across nonpharmacological psychosocial interventions (the main focus of this commentary: French and Kennedy 2018; Green and Garg 2018; Zamzow 2022; Bottema‐Beutel et al. 2022). Anecdotally, there has been a view, persisting in some settings, that rigorous research designs are less possible, necessary, or applicable to psychosocial and educational interventions for individuals with developmental disabilities, such as autism, than they are for medical interventions (Odom et al. 2005; Oliver et al. 2002); or that ethical and practical challenges limit the use of such methods (Poling and Edwards 2014). These views may stem in part from the assumption that such interventions do not pose the same potential adverse risks as medications (although see the section on adverse events below) and are not subject to U.S. Food and Drug Administration (FDA) or equivalent regulations; or that rigorous trial testing of psychosocial and educational interventions would fail to find, or be “sensitive to”, the therapeutic effects that clinicians and families need to see (Reichow et al. 2008; Zamzow 2022). However, much has changed since such views were published. Cumulative intervention science work has established that rigorous clinical trial tests of psychosocial interventions, in autism and beyond, are both possible and ethical, and can show effectiveness (Skivington et al. 2021). Methodological innovations in both design and analysis can address previous concerns about heterogeneity and complexity, indeed showing the powerful potential of the RCT in just such situations (Emsley et al. 2011; Dunn et al. 2015); and mechanistic trials can also identify the key active processes and procedures in particular interventions in a way that can best inform effective yet personalized therapy in complex clinical contexts (Marchette and Weisz 2017). This links to the increasing recent adoption by journals of CONSORT reporting requirements (Hopewell et al. 2025). This is a major advance; showing that the field can and does produce reliable evidence for practice, to reassure funders and service users.

Nevertheless, narrative and systematic reviews of autism intervention research (Green and Garg 2018; Sandbank et al. 2020, 2023) show how risks of bias resulting from poor design can significantly inflate treatment effect estimates (Type 1 error). The quality of trial reporting has also emerged as important to concerns on ‘reproducibility’ in psychological and health care research generally and as a safeguard against misleading clinical claims or investigator conflicts of interest (Ioannidis 2005, 2008). Ethical and equality considerations thus now mandate that the use of rigorous research standards should apply to *all* intervention research (Dawson and Fletcher‐Watson 2022). Without such standards, there is a risk that confidence in the overall results from autism intervention science will be eroded and thus underplay the real gains in evidence and reliable safe interventions for autistic people that have been made.

In this context, a panel and a special interest group (SIG) met at the International Society for Autism Research (INSAR) Annual Meeting in 2023 and 2024 to discuss potential solutions to promote ethical practices, transparency, and rigorous autism intervention science (see Table 1 for summary of mandatory and recommended practices). This group involved autistic people, senior clinical scientists, clinicians, and researchers at all career stages. This commentary is the product of these meetings. Below, we (a) summarize existing challenges associated with poor trial reporting in autism journals, (b) give an overview of the challenges faced when navigating publication standards, (c) suggest recommended and necessary practices for autism journals (see Table 1), and (d) suggest education initiatives and efforts towards an inclusive and community‐based approach to improving scientific reporting standards.

**TABLE 1:** Suggestions for mandatory and recommended practices.

Types of practices	Mandated	Recommended
Registration practices		
Trial pre‐registration^a^ Identification of trial design	X	
Registered final statistical analysis plan with link in paper text (e.g., registered on Open Science Framework [OSF], osf.io)	X	
Prior published protocol paper		X
Submission of registered reports (e.g., *Autism* and *Research in Autism*)		X
Trial reporting standards		
CONSORT 2025 reporting and checklist, appropriate to the design used, in Supporting Information^b^	X	
Identified methodologist/statistician on manuscript (*If not present*, authors should provide an explanation of who supervised the analysis).	X	
Identification of independent oversight (e.g., Data Monitoring and Ethics Committee (DMEC), Data and Safety Monitoring Board (DSMB), or Clinical Trials Unit (CTU) or another appropriate committee)	X	
Possibility of data access (Note caveats around fully anonymous data types with participant permission and with access safeguards)	X	
Information on expected treatment mechanisms is included with RCT reporting Information about adverse event monitoring and reporting^c^	X	X
Additional journal practices		
A culture of journal counter‐commentaries		X
Empirically‐based consideration of masked review practices for trial reports.		X
A RCT registry to disseminate packaged interventions for other groups to study to reduce COIs		X

^a^ Trial pre‐registration (i.e., before the first visit with the first participant) websites include clinicaltrials.gov (USA), ISRCTN registry (UK; https://www.isrctn.com/), Pan African Clinical Trials Register (PACTR; https://pactr.samrc.ac.za/), and the International Clinical Trials Registry Platform (ICTRP; https://www.who.int/clinical‐trials‐registry‐platform) from the World Health Organization (WHO), among other Primary Registries in the WHO Registry Network (https://www.who.int/clinical‐trials‐registry‐platform/network/primary‐registries).

^b^ CONSORT reporting guidelines (https://www.equator‐network.org/reporting‐guidelines/consort/) and extensions (e.g., CONSORT extension for reporting N‐of‐1 trials: https://www.equator‐network.org/reporting‐guidelines/consort‐cent/; CONSORT extension to randomized pilot and feasibility trials: https://www.equator‐network.org/reporting‐guidelines/consort‐2010‐statement‐extension‐to‐randomised‐pilot‐and‐feasibility‐trials/). See Hopewell et al. (2025) for updated guidelines.

^c^ See Lodewyk et al. (2023) for a list of example events from pediatric psychosocial interventions. See Dawson and Fletcher‐Watson (2022) for information on opportunity costs.

## 2 Current Trial and Conflict of Interest Reporting in Autism‐Focused Journals

### 2.1 Consequences of Poor Trial Reporting

Risks of bias are study design and reporting flaws which threaten to inflate resulting estimates of intervention efficacy (Boutron et al. 2022). Risks of bias particularly relevant to nonpharmacological autism intervention research include: (a) selection bias related to inadequate random assignment and allocation concealment, (b) detection bias related to inadequate masking of assessors to participant group assignment, (c) placebo‐by‐proxy bias (Grelotti and Kaptchuk 2011) due to the use of unmasked caregivers as assessors (who are aware of group assignment and may be personally invested in the trial's outcome), and (d) attrition bias related to substantial and differential attrition of participants over the course of the study (Higgins et al. 2020).

In a recent comprehensive systematic review and meta‐analysis of all nonpharmacological interventions designed to support young autistic children (Project AIM), risks of bias were evaluated in a dataset of 289 reports (Sandbank et al. 2023). These reports were identified through a rigorous database search and outreach to grant recipients. They were independently screened and coded by trained research team members using standardized procedures to ensure consistency and minimize bias. Nearly a third (31%) of trials were at risk of selection bias due to the lack of random assignment of participants to intervention arms. Regarding detection bias, nearly two‐thirds (65.2%) of all effects were threatened by high risk of bias due to the absence of masked assessors. Only 14.8% of effects were threatened by substantial and uncontrolled attrition. The consequence of inadequate control of these risks of bias related to study design is inflated intervention effect estimates. Further analyses in Project AIM showed that intervention efficacy estimates become increasingly smaller in magnitude as effects from low‐quality studies are removed.

A subsequent Project AIM investigation examined the extent to which poor reporting practices threaten conclusions about evidence‐based nonpharmacological early autism interventions (Sandbank et al. 2024). These practices include failure to report registered trials after completion, potentially due to reluctance to share null or negative findings. There is also evidence of inadequate reporting and data sharing, which prevents inclusion in meta‐analysis. Selective reporting practices also abound, including failure to report all measured outcomes, reporting outcomes as primary that were registered as secondary or vice versa, reporting data at unplanned assessment points, or changing analysis methods to promote results that seem more favorable. The potential downstream consequence of poorly enforced design and reporting standards for autism intervention research is a less robust evidence base available for systematic reviews and to inform practice recommendations set by guiding clinical organizations.

### 2.2 Adverse Events

In psychosocial healthcare intervention research, more broadly, there has been insufficient reporting on the occurrence of adverse events. Providing this information is necessary to allow practitioners and families to learn positively from trials, and to weigh the potential for positive intervention effects with the potential for negative side effects, to ensure informed decision making (Dawson and Fletcher‐Watson 2022). Such practices include failing to design and implement active adverse event monitoring plans, failure to collect complete information about their occurrence, and failure to report or fully report information (Ioannidis 2009). For example, a review of the 289 reports of early intervention autism research included in Project AIM found that only 10% of these reports mentioned adverse events, and only 8 of these described monitoring procedures for tracking whether adverse events had occurred (Sandbank et al. 2023).

### 2.3 Conflicts of Interest (COI)

Systematic evaluations of autism intervention research have also shown that COIs are widespread in autism intervention research, but are rarely disclosed (Bottema‐Beutel et al. 2021; Bottema‐Beutel and Crowley 2021). COIs occur in situations where researchers stand to gain from showing positive findings, such as when they study interventions they developed, are employed as intervention providers, or receive speaker fees for presentations related to their research. There is some evidence that COIs are associated with inflated effect sizes (Bottema‐Beutel et al. 2021). Failure to disclose COIs is often in violation of journal policies. In a study of autism intervention research published in Applied Behavior Analysis journals over a one‐year period (September 2019–September 2020), 100% of the articles in three out of six journals included in the study that required COI statements included false statements that researchers had no COIs (Bottema‐Beutel and Crowley 2021). The COI in question was that researchers were employed as providers of interventions related to the intervention being investigated or were employed as intervention consultants, a COI that was explicitly defined as requiring disclosure in journal submission guidelines (Dawson and Fletcher‐Watson 2021).

## 3 Introduction of Mandatory and Recommended Practices for Autism Journals

This commentary proposes that all autism‐focused journals introduce guidance to improve the transparency of trial requirements and submission quality. It is proposed that this guidance could distinguish between mandated (e.g., publication of trial registration, protocols, and analysis plans, full CONSORT reporting standards) and recommended (e.g., protocol papers) practices (see Table 1). Here, we provide further support for such an introduction.

### 3.1 Trial Reporting Standards

We suggest that standardized reporting criteria in autism‐focused journals, alongside guidance for peer reviewers, could be the quickest driver for increased transparently reported evidence in the field. It would also improve the efficiency of the review and editorial decision‐making process. There is, usefully to facilitate this, a new major CONSORT 2025 revision statement (Hopewell et al. 2025), which updates guidance and reporting recommendations in the light of many contemporary issues. Nearly half of early childhood autism trials are published in just five autism‐focused journals (Sandbank et al. 2020). Whilst four out of these five journals have now begun to mandate CONSORT reporting procedures, enforcement of these requirements in review is uncertain, since, historically, reviews mentioned above have found inconsistent adherence to reporting requirements. Even when journals have robust reporting requirements for authors, these can be challenging to implement consistently by a large and diverse group of editors, when reviewers have varying expectations of what constitutes robust reporting, and when authors may position their work as a “pilot” in order to avoid certain expectations [Fletcher‐Watson, personal communication]. Further, a minority of larger trials are published in high‐impact general medical journals (e.g., NEJM, JAMA, Lancet journals), which enforce detailed requirements for trial reporting. This creates unevenness in the resulting autism intervention science literature.

Well‐powered randomized controlled trials (RCTs) with designs minimizing risks of bias are the gold standard international method for best establishing efficacy and generalizable effectiveness within intervention science and should be taken as the key evidence in our field. However, formal pilot studies, quasi‐experimental uncontrolled observational designs, single‐case designs, and qualitative methods are also valuable when used at different stages of intervention development or testing, and adhere to design standards and reporting appropriate for each method (see Vivanti and Messinger 2021, and Vivanti 2022 for a brief history in autism research). The key issue is the nature of inferences that can reasonably be drawn from each design, and that reviewers and editors are able to spot when unwarranted inferences are made. Single case designs can have value in identifying the immediate local effects of a focused intervention for an individual, but lack capacity to determine whether such effects generalize to a wide range of contexts and reflect broader, more distal developmental improvement (Ledford et al. 2018; Sandbank et al. 2020, 2021). Pilot studies are invaluable to establish feasibility and acceptability, but are not designed to report efficacy or effectiveness and thus should not postulate (or be asked by reviewers to postulate) such conclusions (Eldridge et al. 2016). Helpfully, CONSORT guidelines exist for a range of intervention trial reports, including the CONSORT extension for reporting N‐of‐1 trials (i.e., CENT; Vohra et al. 2015) and for pilot and feasibility trials (Eldridge et al. 2016). For more nuanced trial designs (i.e., SMARTs), journals can adopt recommended reporting guides to ensure consistency between reviewers (Hampton et al. 2023; Hopewell et al. 2025).

The SIG therefore recommends that a rigorous approach to linking study design with appropriate inferences be included in journal reviewer guidance, as well as corresponding education initiatives to facilitate its uptake. We are not advocating constraint on the particular type of trial to be conducted, but mandating that *each trial method is reported transparently and appropriately for its type and that the Abstract and Discussion sections are clear on what can and cannot be inferred from the design used*. These improvements in themselves would have huge benefits for the field. Other emerging concerns are: 1) the selective reporting of moderation or mediation analyses in trials with null effects, presented in such a way as to suggest efficacy while minimizing, or not reporting, the null result itself; and 2) presenting null results of active treatment control trials as evidence that both interventions were equally effective. Reviewers need to be alert to these trends, which would be mitigated by mandated reporting of primary outcomes prior to mediation studies, and pre‐registration of mediation analyses. Appropriate use of these standards could then make null results more meaningful and informative, and encourage journals to publish them. Guidelines supportive of change in response to this file drawer problem (Transparency and Openness Promotion [TOP]; Nosek et al. 2015) have been endorsed by the American Psychological Association (APA), and promote the use of registered reports (in which publication of well‐designed trials is guaranteed independent of results).

## 4 Barriers and Opportunities From an Editorial Perspective

### 4.1 Knowledge and Implementation of Reporting Standards

Even where reporting standards are expected and clearly outlined within the submission guidelines, such as requiring pre‐registration and a CONSORT flowchart and checklist for clinical trials, editors and reviewers may not always be vigilant about requesting these materials or fully understand how to evaluate the information provided. Studies described as an “evaluation,” “pilot,” or “feasibility study” may not be subject to equivalent scrutiny, something that could be helped by obligatory drop‐down menus nominating study design options or templated forms, based upon the list of Equator categories (https://www.equator‐network.org/reporting‐guidelines‐study‐design/clinical‐trials‐experimental‐studies/). The recently updated CONSORT guidance (Hopewell et al. 2025) can provide additional clarity in this regard.

### 4.2 Space Limits

Space limits in journals can seem challenging for comprehensive trial reporting; compounded when authors realize reporting expectations for trials only at the point of submission. However, this rationale does not justify partial reporting. Improved initial editorial signposting on submission, better use of Supporting Information, and prior publication of referenced ‘Protocol’ papers, all make concise but complete reporting possible.

### 4.3 Reviewer Expertise

Locating scholars with the necessary expertise to conduct high‐quality reviews of manuscripts reporting clinical trial outcomes can be challenging in the current peer review system. Reviewers currently give their time for free as a service to the scientific community, but within the broader context of a publication system that is often criticized as exploiting such service. In this context, expecting reviewers to complete additional training before they have the privilege of reviewing for a journal seems unreasonable. Instead, it would be more practical to offer official credentialed training to scholars seeking to conduct clinical trials, so that they may simultaneously increase their professional capacities while widening the potential reviewer pool. INSAR could play a crucial role in this, as discussed below. Overall, we want to emphasize a shared responsibility between the roles of Publisher, Editor, Reviewer, and Author in raising the standards for intervention science reporting; at the service of science and the autistic community generally.

### 4.4 Masked Reviews

Arguments in favor of masked reviewing include equity and mitigating reviewer bias. However, anonymization of a trial report makes it impossible for a reviewer to check trial registration, OSF details, or protocol papers, all of which provide vital necessary evidence to inform review accuracy. It is also contrary to trends in open science reporting and undermines the value of prior registration. The SIG reflected these differing views and advocated that the issue is important enough to be seriously and critically considered by Editors. We advocate that Journals ensure procedures that *always* enable Reviewers and/or Editors to cross‐check in detail what is written in a trial report against the relevant trial registration, oversight, and protocol information. In the case of masked reviewing, this would include consideration of: (a) whether full masked registration and oversight information could be provided for review; (b) whether reviewers can guess trial authors despite masking; (c) whether other measures, separate to masking, could be taken to mitigate reviewer conflicts of interest or bias.

## 5 Education Initiatives

As the field of autism research expands, it is an opportune time to establish support systems for ECRs, established researchers or clinicians pivoting to intervention research, and those across low‐ and middle‐income settings to ensure researchers learn and implement best practices. INSAR itself can develop training modules (e.g., how to report pilot study criteria, monitoring and reporting adverse events, COI reporting) and host open access materials or templates that can help support best practices. This could be done through the existing INSAR Scientific Standards and Training Committees, which could leverage the INSAR annual meeting to hold workshops that would support the uptake and dissemination of best practices and reporting standards for levels of evaluation (https://www.equator‐network.org/). The important and well‐attended virtual INSAR institutes can also support capacity building and are accessible globally (key to meeting global inequalities of opportunity in this area). The creation of formal training modules (e.g., “INSAR‐ Clinical Trial Reviewer”) could ideally allow ECRs both to expand their professional capacities and to showcase added skills on their curriculum vitae. These initiatives should lean towards understanding the contextual need for conducting research in real‐world settings, such as in busy tertiary services where the capacity for the rigor of RCTs may not be available. In such cases, transparency and candidness in reporting the limitations and potential biases will ensure that such studies are evaluated on their true merit. Reporting standards are more important than ever in cases where adequacy in trial administration is not achieved. As above, we need more work disseminating internationally expected standards of research design, linked to the inferences that can be drawn from each.

We recommend that autism‐focused journals improve the peer review process through the provision of training and support materials to facilitate comprehensive reviewer feedback aligned with transparent guidelines for high‐quality trial reporting. For example, journals may provide optional training videos with advice for reviewers, require reviewers to complete a checklist that guarantees trial reporting consistency, and/or provide a structured template for review comments to authors. SIG members also offered a variety of suggestions to incentivize reviewers, such as payment, acknowledgment of reviewers in the manuscript, the creation of training modules that facilitate certification that could be endorsed on a CV, and the use of Publons to showcase reviewer contributions.

## 6 Community Involvement

Historically, autism research and practice have been relatively slow in developing participatory work with the autistic community, as well as other community members (e.g., caregivers, service users; Fletcher‐Watson et al. 2019). However, recent years have seen increasing impetus towards this, stimulated by advocacy from the neurodiversity movement and wider recognition of this need (Bertilsdotter Rosqvist et al. 2019; Botha and Cage 2022; Chown et al. 2017; Leadbitter et al. 2021; Milton and Bracher 2013). We highlight the importance of moving towards co‐design with stakeholders and the autistic community for both intervention types and trial design, including measurement design and selection (See Schiltz et al. 2024 for a commentary on patient‐reported outcome measures), as community members may inform priority outcomes for measurement to align with community values (e.g., the Autistic Adults and other Stakeholders Engage Together (AASET) Community Council, Benevides et al. 2020). To do this, practice guidance for researchers and community partners in developing skill sets necessary to contribute meaningfully, protecting autistic individuals from harm, and giving people credit for their contributions would be valuable (see Cullingham et al. 2024, Lee et al. 2024 and Shore and Benevides 2018 for examples). INSAR may be an avenue to support the development and dissemination of these training efforts. Autistic people and their loved ones deserve every right to the best standards of evidence‐based care and to comprehensive information to make choices about their care, through an intervention evidence base that is robust and enables them and their clinicians to make informed decisions.

## 7 Conclusion

This commentary summarizes recommendations to improve autism intervention research from the perspective of autistic people, senior clinical scientists, clinicians, and researchers who attended SIG meetings at the 2023 and 2024 INSAR Annual Meetings. We advocate for maximizing the quality and reporting standards of autism intervention science through more transparent and comprehensive trial registration (e.g., trial pre‐registration, registration of statistical analysis plan) and reporting (e.g., CONSORT reporting, identification of a methodologist/statistician, identification of independent oversight, and possibility of data access) for studies of psychosocial interventions across all stages (e.g., pilot research, RCTs, implementation studies). However, the responsibility to improve our intervention science standards should be a partnership, and there is a crucial role for systemic change in autism‐focused journals to encourage and support these efforts; such as improved submission guidelines and procedures, providing templates and training opportunities to intervention researchers and reviewers to ensure that studies adhere to best practice recommendations, and reviewer incentives. ECRs, students and trainees, autistic people and other stakeholders, and clinicians may also benefit from educational support to encourage best practices in intervention research and in peer review; developed perhaps in partnership between INSAR Scientific Standards and Training Committees, annual INSAR Institutes, and journals themselves. To reach this new bar for autism intervention science, we will need collaboration across researchers, clinicians, journals, and the autism community to improve our evidence base and, ultimately, to provide effective supports for autistic people and their families around the world.

## Conflicts of Interest

S.L. was formerly affiliated with an entity that trained students to become Board Certified Behavior Analysts and provided Early Intensive Behavioral Intervention. C.B.K. received funding as a graduate student to be an interventionist in the US National Institute of Child Health and Human Development (NICHD) and DoD‐funded clinical trials for autistic transition‐age youth. M.S. has received fees for presenting research findings in invited talks and providing expert evidence related to the efficacy of early intervention in court hearings. K.B.‐B. has previously received fees for consulting with school districts on intervention practices for autistic children and to discuss her work on research quality, adverse events, and researcher conflicts of interest as they pertain to autism intervention research. She receives royalties for Clinical Guide to Early Interventions for Children with Autism, published by Springer. S.F.‐W. is editor in chief of the journal Autism and receives an annual stipend for that role. M.E.A. is on the board of two not‐for‐profit organizations providing training and courses and one providing school services.DD is Editor In Chief for the journal Research in Developmental Disabilities and Research in Neurodiversity. A.B. has previously received fees for consulting with community clinics on support services and has delivered PD and taught courses related to autism support services. She has also received research funding to explore the impact of interventions used in community practice. T.C. has served as a paid consultant to F. Hoffmann‐La Roche Ltd. and receives royalties from Sage Publications and Guilford Publications. J.A.E. has received honoraria for presenting on autism research and interventions. A.F. is a PhD candidate at the University of Milano Bicocca, Milan. M.‐M.G. is a certified trainer in ESDM and other therapies (e.g., PACT). L.H.H. is funded by the National Institute on Deafness and Other Communication Disorders (NIDCD). E.S.K. is currently leading the development of an intervention for transition‐age autistic teenagers and young adults with funding from the NICHD. J.Y.S.L. is involved in the development of a parent support program for autistic children (AutInsight) and recently ran a pilot trial for the efficacy of this program. J.S. works on a University of Antwerp R2D2‐MH project facilitating co‐creation between neurodivergent people and neurodiversity researchers. D.S. received funding from the National Institute of Mental Health (NIMH) to assist with research exploring mechanisms of three early intervention models for autism. S.V. has worked on NIH and Institute of Education Sciences (IES) funded studies of early autism intervention, coordinating and providing direct intervention and parent coaching and training providers. G.V. receives royalties from the books “Implementing the Group‐Based Early Start Denver Model for Young Children with Autism” and “Clinical Guide to Early Interventions for Children with Autism.” J.G. has received funding from UK Medical Research Council and National Institute for Health Research (NIHR) and receives director's fees from a not‐for‐profit PACT training company IMPACT (CiC 10902031). G.D., S.M.F., S.H., and C.W. declare no conflicts of interest.

## Data Availability

Data quoted in this Commentary is available from the associated cited references.

## References

[aur70126-bib-0001] Benevides, T. W. , S. M. Shore , K. Palmer , et al. 2020. “Listening to the Autistic Voice: Mental Health Priorities to Guide Research and Practice in Autism From a Stakeholder‐Driven Project.” Autism 24, no. 4: 822–833. 10.1177/1362361320908410. 32429818 PMC7787673

[aur70126-bib-0002] Bertilsdotter Rosqvist, H. , M. Kourti , D. Jackscon‐Perry , et al. 2019. “Doing It Differently: Emancipatory Autism Studies Within a Neurodiverse Academic Space.” Disability & Society 34, no. 7–8: 1082–1101. 10.1080/09687599.2019.1603102.

[aur70126-bib-0003] Botha, M. , and E. Cage . 2022. “‘Autism Research Is in Crisis’: A Mixed Method Study of Researcher's Constructions of Autistic People and Autism Research.” Frontiers in Psychology 13: 1050897. 36506950 10.3389/fpsyg.2022.1050897 PMC9730396

[aur70126-bib-0004] Bottema‐Beutel, K. , and S. Crowley . 2021. “Pervasive Undisclosed Conflicts of Interest in Applied Behavior Analysis Autism Literature.” Frontiers in Psychology 12: 676303. 10.3389/fpsyg.2021.676303. 34025538 PMC8131529

[aur70126-bib-0005] Bottema‐Beutel, K. , S. Crowley , M. Sandbank , and T. G. Woynaroski . 2021. “Research Review: Conflicts of Interest (COIs) in Autism Early Intervention Research: A Meta‐Analysis of COI Influences on Intervention Effects.” Journal of Child Psychology and Psychiatry, and Allied Disciplines 62, no. 1: 5–15. 10.1111/jcpp.13249. 32353179 PMC7606324

[aur70126-bib-0006] Bottema‐Beutel, K. , S. C. LaPoint , S. Y. Kim , S. Mohiuddin , Q. Yu , and R. McKinnon . 2022. “An Evaluation of Intervention Research for Transition‐Age Autistic Youth.” Autism 27, no. 4: 890–904. 10.1177/13623613221128761. 36189778

[aur70126-bib-0007] Boutron, I. , M. J. Page , J. P. T. Higgins , D. G. Altman , A. Lundh , and A. Hróbjartsson . 2022. “Chapter 7: Considering Bias and Conflicts of Interest Among the Included Studies.” In Cochrane Handbook for Systematic Reviews of Interventions (Version 6.5), edited by J. P. T. Higgins , J. Thomas , J. Chandler , et al. Cochrane. https://www.cochrane.org/handbook.

[aur70126-bib-0008] Chown, N. , J. Robinson , L. Beardon , et al. 2017. “Improving Research About Us, With Us: A Draft Framework for Inclusive Autism Research.” Disability & Society 32, no. 5: 720–734. 10.1080/09687599.2017.1320273.

[aur70126-bib-0009] Cullingham, T. , U. Rennard , C. Creswell , et al. 2024. “The Process of Co‐Design for a New Anxiety Intervention for Autistic Children.” JCPP Advances 5: e12255. 10.1002/jcv2.12255. 40519955 PMC12159306

[aur70126-bib-0010] Dawson, M. , and S. Fletcher‐Watson . 2021. “Commentary: What Conflicts of Interest Tell Us About Autism Intervention Research—A Commentary on Bottema‐Beutel Et al.(2020).” Journal of Child Psychology and Psychiatry 62, no. 1: 16–18. 32845011 10.1111/jcpp.13315

[aur70126-bib-0011] Dawson, M. , and S. Fletcher‐Watson . 2022. “When Autism Researchers Disregard Harms: A Commentary.” Autism 26, no. 2: 564–566. 34291651 10.1177/13623613211031403 PMC8814944

[aur70126-bib-0012] Dunn, G. , R. Emsley , H. Liu , et al. 2015. “Evaluation and Validation of Social and Psychological Markers in Randomised Trials of Complex Interventions in Mental Health: A Methodological Research Programme.” Health Technology Assessment 19, no. 93: 1–116. 10.3310/hta19930. PMC4781463 26560448

[aur70126-bib-0013] Eldridge, S. M. , C. L. Chan , M. J. Campbell , et al. 2016. “CONSORT 2010 Statement: Extension to Randomised Pilot and Feasibility Trials.” Pilot and Feasibility Studies 2: 64. 10.1186/s40814-016-0105-8. 27965879 PMC5154046

[aur70126-bib-0014] Emsley, R. A. , J. Green , and G. Dunn . 2011. “Designing Trials of Complex Interventions for Efficacy and Mechanisms Evaluation'.” Trials 12: A143. 10.1186/1745-6215-12-S1-A143.

[aur70126-bib-0015] Fletcher‐Watson, S. , J. Adams , K. Brook , et al. 2019. “Making the Future Together: Shaping Autism Research Through Meaningful Participation.” Autism 23, no. 4: 943–953. 10.1177/1362361318786721. 30095277 PMC6512245

[aur70126-bib-0016] French, L. , and E. M. M. Kennedy . 2018. “Annual Research Review: Early Intervention for Infants and Young Children With, or At‐Risk of, Autism Spectrum Disorder: A Systematic Review.” Journal of Child Psychology and Psychiatry 59, no. 4: 444–456. 10.1111/jcpp.12828. 29052838

[aur70126-bib-0017] Green, J. , and S. Garg . 2018. “The State of Autism Intervention Science: Process, Target Psychological and Biological Mechanisms and Future Prospects.” Journal of Child Psychology and Psychiatry, and Allied Disciplines 59, no. 4: 424–443. 10.1111/jcpp.12892. 29574740

[aur70126-bib-0018] Grelotti, D. J. , and T. J. Kaptchuk . 2011. “Placebo by Proxy.” BMJ 343: d4345. 10.1136/bmj.d4345. 21835868 PMC3230083

[aur70126-bib-0019] Hampton, L. H. , J. C. Chow , B. H. Bhat , and G. Roberts . 2023. “Reporting and Design Considerations for SMART Behavioral Science Research.” Educational Psychology Review 35, no. 4: 117.

[aur70126-bib-0020] Higgins, J. P. T. , J. Savović , M. J. Page , R. G. Elbers , and J. A. C. Sterne . 2020. “Bias due to Missing Outcome Data.” In Cochrane Handbook for Systematic Reviews of Interventions (Version 6.5), edited by J. P. T. Higgins , J. Thomas , J. Chandler , et al. Cochrane 2024. https://cochrane.org/handbook.

[aur70126-bib-0021] Hopewell, S. , A.‐W. Chan , G. S. Collins , et al. 2025. “CONSORT 2025 Statement: Updated Guideline for Reporting Randomised Trials.” BMJ 389: e081123. 10.1136/bmj-2024-081123. 40228833 PMC11995449

[aur70126-bib-0022] Ioannidis, J. P. 2005. “Why Most Published Research Findings Are False.” PLoS Medicine 2, no. 8: e124. 10.1371/journal.pmed.0020124. 16060722 PMC1182327

[aur70126-bib-0023] Ioannidis, J. P. 2008. “Why Most Discovered True Associations Are Inflated.” Epidemiology 19, no. 5: 640–648. 10.1097/EDE.0b013e31818131e7. 18633328

[aur70126-bib-0024] Ioannidis, J. P. 2009. “Adverse Events in Randomized Trials: Neglected, Restricted, Distorted, and Silenced.” Archives of Internal Medicine 169, no. 19: 1737–1739. 10.1001/archinternmed.2009.313. 19858427

[aur70126-bib-0025] Leadbitter, K. , K. L. Buckle , C. Ellis , and M. Dekker . 2021. “Autistic Self‐Advocacy and the Neurodiversity Movement: Implications for Autism Early Intervention Research and Practice.” Frontiers in Psychology 12: 635690. 10.3389/fpsyg.2021.635690. 33912110 PMC8075160

[aur70126-bib-0026] Ledford, J. R. , J. D. Lane , and D. L. Gast . 2018. “Dependent Variables, Measurement, and Reliability.” In Single Case Research Methodology, 97–131. Routledge.

[aur70126-bib-0027] Lee, J. , M. G. Onaiwu , K. Bottema‐Beutel , et al. 2024. “Strategies for Community Engagement With Autistic Adults and Caregivers in Early Intervention Autism Research.” https://bpb‐us‐e1.wpmucdn.com/sites.northwestern.edu/dist/0/8134/files/2024/09/EngagementStrategies9.9.pdf.

[aur70126-bib-0028] Lodewyk, K. , A. Bagnell , D. B. Courtney , and A. S. Newton . 2023. “Adverse Event Monitoring and Reporting in Studies of Pediatric Psychosocial Interventions: A Systematic Review.” Child and Adolescent Mental Health 28, no. 3: 425–437. 37463769 10.1111/camh.12661

[aur70126-bib-0029] Marchette, L. K. , and J. R. Weisz . 2017. “Practitioner Review: Empirical Evolution of Youth Psychotherapy Toward Transdiagnostic Approaches.” Journal of Child Psychology and Psychiatry 58, no. 9: 970–984. 10.1111/jcpp.12747. 28548291

[aur70126-bib-0030] Milton, D. , and M. Bracher . 2013. “Autistics Speak but Are They Heard?” A Journal of the BSA MedSoc Group 7, no. 2: 61–69.

[aur70126-bib-0031] Nosek, B. A. , G. Alter , G. C. Banks , et al. 2015. “Promoting an Open Research Culture.” Science 348, no. 6242: 1422–1425. 10.1126/science.aab2374. 26113702 PMC4550299

[aur70126-bib-0032] Odom, S. L. , E. Brantlinger , R. Gersten , R. H. Horner , B. Thompson , and K. R. Harris . 2005. “Research in Special Education: Scientific Methods and Evidence‐Based Practices.” Exceptional Children 71, no. 2: 137–148.

[aur70126-bib-0033] Oliver, P. C. , J. Piachaud , J. Done , A. Regan , S. Cooray , and P. Tyrer . 2002. “Difficulties in Conducting a Randomized Controlled Trial of Health Service Interventions in Intellectual Disability: Implications for Evidence‐Based Practice.” Journal of Intellectual Disability Research 46: 340–345. 12000585 10.1046/j.1365-2788.2002.00408.x

[aur70126-bib-0034] Poling, A. , and T. L. Edwards . 2014. “Ethical Issues in Early Intervention.” In Handbook of Early Intervention for Autism Spectrum Disorders: Research, Policy, and Practice, 141–164. Springer.

[aur70126-bib-0035] Reichow, B. , F. R. Volkmar , and D. V. Cicchetti . 2008. “Development of the Evaluative Method for Evaluating and Determining Evidence‐Based Practices in Autism.” Journal of Autism and Developmental Disorders 38: 1311–1319. 10.1007/s10803-007-0517-7. 18095149

[aur70126-bib-0036] Sandbank, M. , K. Bottema‐Beutel , S. Crowley , et al. 2020. “Project AIM: Autism Intervention Meta‐Analysis for Studies of Young Children.” Psychological Bulletin 146, no. 1: 1–29. 10.1037/bul0000215. 31763860 PMC8783568

[aur70126-bib-0037] Sandbank, M. , K. Bottema‐Beutel , S. C. LaPoint , et al. 2023. “Autism Intervention Meta‐Analysis of Early Childhood Studies (Project AIM): Updated Systematic Review and Secondary Analysis.” BMJ (Clinical Research Ed.) 383: e076733. 10.1136/bmj-2023-076733. PMC10644209 37963634

[aur70126-bib-0038] Sandbank, M. , K. Bottema‐Beutel , Y. Syu , N. Caldwell , J. I. Feldman , and T. Woynaroski . 2024. “Evidence‐B(i)ased Practice: Selective and Inadequate Reporting in Early Childhood Autism Intervention Research.” Autism 28, no. 8: 1889–1901. 10.1177/13623613241231624. 38345030 PMC11301951

[aur70126-bib-0039] Sandbank, M. , J. Chow , K. Bottema‐Beutel , and T. Woynaroski . 2021. “Evaluating Intervention Effects in Light of Boundedness and Proximity.” Autism Research 10: 1536–1542. 10.1002/aur.2527. PMC9552805 33943022

[aur70126-bib-0040] Schiltz, H. K. , Z. J. Williams , S. Zheng , et al. 2024. “Measurement Matters: A Commentary on the State of the Science on Patient Reported Outcome Measures (PROMs) in Autism Research.” Autism Research 17, no. 4: 690–701. 10.1002/aur.3114. 38429884

[aur70126-bib-0041] Shore, S. , and T. Benevides . 2018. “Autistic Adults and Other Stakeholders Engage Together Engagement & Compensation Guide.” https://www.pcori.org/sites/default/files/Engagement‐Guide‐as‐of‐122018‐2.1.pdf.

[aur70126-bib-0042] Skivington, K. , L. Matthews , S. A. Simpson , et al. 2021. “A New Framework for Developing and Evaluating Complex Interventions: Update of Medical Research Council Guidance.” BMJ 374: n2061. 10.1136/bmj.n2061. 34593508 PMC8482308

[aur70126-bib-0043] Vivanti, G. 2022. “What Does It Mean for an Autism Intervention to Be Evidence Based?” Autism Research 15, no. 10: 1787–1793. 10.1002/aur.2792. 36065991

[aur70126-bib-0044] Vivanti, G. , and D. S. Messinger . 2021. “Theories of Autism and Autism Treatment From the DSM III Through the Present and Beyond: Impact on Research and Practice.” Journal of Autism and Developmental Disorders 51, no. 12: 4309–4320. 33491120 10.1007/s10803-021-04887-z

[aur70126-bib-0045] Vohra, S. , L. Shamseer , M. Sampson , et al. 2015. “CONSORT Extension for Reporting N‐Of‐1 Trials (CENT) 2015 Statement.” BMJ (Clinical Research Ed.) 350: h1738. 10.1136/bmj.h1738. 25976398

[aur70126-bib-0046] Zamzow, R. 2022. “Why Autism Therapies Have an Evidence Problem.” Thetransmitter.org. https://www.thetransmitter.org/spectrum/why‐autism‐therapies‐have‐an‐evidence‐problem/.

